# Tumor-derived circulating exosomal miR-342-5p and miR-574-5p as promising diagnostic biomarkers for early-stage Lung Adenocarcinoma

**DOI:** 10.7150/ijms.43500

**Published:** 2020-06-06

**Authors:** Zhijun Han, Yangyang Li, Jian Zhang, Chongye Guo, Qian Li, Xin Zhang, Yongqing Lan, Wenbin Gu, Zhikai Xing, Liang Liang, Meng Li, Shuangli Mi

**Affiliations:** 1 Department of Thoracic Surgery, Peking Union Medical College Hospital (PUMCH), Chinese Academy of Medical Sciences & Peking Union Medical College (CAMS & PUMC), Beijing, 100730, P.R. China.; 2 Key Laboratory of Genomic and Precision Medicine, Beijing Institute of Genomics, Chinese Academy of Sciences, China National Center for Bioinformation, Beijing, 100101, P.R. China.; 3 University of Chinese Academy of Sciences, Beijing, 100049, P.R. China.

**Keywords:** exosomal miRNA, exosome, diagnostic biomarker, lung adenocarcinoma

## Abstract

Lung cancer has been the leading cause of cancer morbidity and mortality in recent years. Most lung cancers are often asymptomatic until advanced or metastatic stage. Therefore, looking for the diagnostic biomarker for early-stage lung cancer is quite significant. Circulating exosomal microRNAs (miRNAs) have been reported to be the diagnostic and prognostic markers of various cancers. Here, we obtained circulating exosomal miRNA repertoires of 7 early-stage lung adenocarcinoma patients including pre-operation and post-operation (LA-pre and LA-post) and 7 heathy controls (HCs) by next generation sequence (NGS) and selected miR-342-5p, miR-574-5p and miR-222-3p to validate in ampliative samples by reverse transcription-quantitative PCR (RT-qPCR). Circulating exosomal miR-342-5p, miR-574-5p and miR-222-3p not only significantly elevated in LA patients (n = 56) compared with HCs (n = 40), but also significantly decreased after tumor resection when analyzed 51 paired pre- and post-operation samples. Furthermore, miR-342-5p and miR-574-5p, but not miR-222-3p, had a significantly elevated expression level in carcinoma tissue compared with adjacent non-cancerous tissue (n = 8). The receiver operating characteristic (ROC) curve showed the area under the curve (AUC) of combined miR-342-5p and miR-574-5p was 0.813 (95% CI: 0.7249 to 0.9009) with sensitivity and specificity of 80.0% and 73.2% respectively. In summary, circulating exosomal miR-342-5p and miR-574-5p have potential to serve as novel diagnostic biomarkers for early-stage LA.

## Introduction

Lung cancer remains the leading cause of cancer morbidity and mortality 1. In spite of advance in the diagnosis and current molecular targeted therapies for lung cancer, the predicted 5-year survival rate is still quite low (18.4%) 1. In all lung cancer cases, non-small cell lung cancer (NSCLC) accounts for 85%, and NSCLC is divided into LA and lung squamous cell carcinoma (LSCC) with each one accounting for approximately 30% to 50% 2. There are many early identification techniques for lung cancer clinical diagnosis such as pneumonocentesis, X-ray, and computerized tomography (CT) 3. Surgical resection with adjuvant chemotherapy is regarded as the optimal treatment of early-stage NSCLC at present 4. However, about 70% of lung cancers are confirmed at advanced or metastatic stage, which delays the treatment 5. The existing serum protein biomarkers, such as carcinoembryonic antigen (CEA) and CYFRA21-1 that have been applied to clinical cancer screening, did not show sufficient sensitivity and specificity 6. After surgical resection, the 5-year survival rate of IA and IIIA stage lung cancer patients are 73% and 23% respectively 7. Thus, looking for the reliable diagnostic biomarkers of early-stage lung cancer is quite urgent and important.

MiRNAs are small non-coding single stranded RNAs with about 22 nucleotides in length, which could mediate mRNAs degradation or translational inhibition 8. The miRNA expression profiles are dysregulated in tumor cells 9, 10, which makes miRNAs as a class of biomarkers for cancers. MiRNAs circulating in blood have been found to be the noninvasive biomarkers of various diseases including lung cancer 11, 12. Exosomes are 30-150 nm in diameter, membrane-enclosed extracellular vesicles (EVs), containing lipids, proteins, and nucleotides 13. MiRNAs are one of the major components of exosomal RNAs, and could be delivered into recipient cells as a kind of regulatory molecules 14. Exosomal miRNAs suspending in body fluids have become ideal biomarkers for the following three reasons: First, exosomes distribute in various available body fluids, such as blood, saliva, urine, breast milk and amniotic fluid 14. Several of these body fluids could be collected by a noninvasive method. Second, the contents of exosomes depend on their original cells 15. Exosomes derived from different kinds of tumor cells have different miRNAs profiles. Third, the bilayer lipid membrane of exosomes protects the contents from degradation, especially for the fragile single stranded RNA 16, 17. MiRNAs in plasma exosomes show a higher stability than plasma miRNAs under different storage conditions 18. Circulating exosomal miRNAs have been reported to distinguish lung cancer patients from healthy individuals. For examples, a panel of three miRNAs (miR-106a-5p, miR-20a-5p and miR-93-5p) was elevated in serum and serum exosomes of male LSCC patients and serum exosomal miR-126 was elevated in NSCLC patients at early- and advanced-stages compared with a control group 19, 20.

To objectively screen circulating exosomal miRNAs as biomarkers, comprehensive study of the miRNAs profiles is necessary. NGS is a talented technology to facilitate nucleotide biomarkers screening extensively. Exosomes in circulatory system are mixed due to different origins, leading to a disturbing non-specificity for biomarker screening. Collecting tissue/organ specific exosomes from peripheral blood could reduce noise, which has been mentioned in a study about screening urinary exosomal miRNAs biomarker of prostate cancer 21. However, this technology has not been well realized so far 22. A recent report regarded EpCAM positive exosomes in plasma as tumor-derived exosomes. By isolating EpCAM positive exosomes in plasma and analyzing miRNA profiles, they found several LA specific and LSCC specific circulating exosomal miRNAs as diagnosis biomarkers of early-stage lung cancer patients 3.

In the present study, we adopted a double compared method to identify tumor-derived circulating exosomal miRNAs as diagnostic biomarkers of early-stage LA by NGS and RT-qPCR. We compared the exosomal miRNAs profiles not only between LA patients and HCs, but also between the LA-pre and the paired LA-post samples from the same LA patient. The overlap of different exosomal miRNAs in these two analyses could indirectly reflect tumor-derived miRNAs 23. With the validation in extended samples, we found that tumor-derived circulating exosomal miR-342-5p and miR-574-5p were promising diagnosis biomarkers for early-stage LA patients. Moreover, we also detected an elevated expression level of both miRNAs in lung carcinoma tissue compared with adjacent non-cancerous tissue.

## Materials and Methods

### Patients and clinical samples

Our study enrolled early-stage (ⅠA/ⅠB) LA patients and advanced-stage (ⅢA) LA patients diagnosed in the Peking Union Medical College Hospital (Beijing, China) between October 2016 and August 2018. The definition of the sample stage is based on the 8^th^ edition of lung cancer TNM grading and confirmed by pathological examination based on the criteria of the World Health Organization. Pre-operation samples were collected the day before surgical resection of the primary tumor. Post-operation samples were collected on the third day after surgical resection of the primary tumor. Peripheral blood was collected in vacuum blood tubes with EDTA and centrifuged at 1,500× g for 15 min at 4 °C within 30 min after sample collection. The supernatant plasma samples were separated and stored at -80 °C. Surgical specimens of primary tumor tissues and adjacent non-cancerous tissues were also obtained. Healthy donors enrolled in our study were healthy volunteers who conducted routine physical examination without lung disease or viral infection. Blood samples of healthy donors were collected as healthy controls. Basic information of all samples used in this study were shown in Table 1.

The work described has been carried out in accordance with The Code of Ethics of the World Medical Association. The study protocol has been approved by the Clinical Research Ethics Committee of the Peking Union Medical College Hospital on human research. The written informed consents were obtained from all the participants.

### Exosome isolation

The exosomes were isolated from the plasma samples by ultracentrifugation. Briefly, approximate 4 ml plasma was thawed on ice and centrifuged at 3000 × g for 10 min to remove possible cell residues. After being filtered with 0.22 μm filter membranes, blood samples were balanced by adding phosphate buffer saline (PBS). Cleared blood samples were centrifuged at 120,000 × g for 10 h using P70AT rotor (Hitachi, Japan). All centrifugal steps were kept at 4 °C. Exosome fractions were resuspended in 250 μL PBS for the following RNA extraction.

### Exosome particle size analysis

Exosome pellets were fixed and examined using a Hitachi H-7650 transmission electron microscopy (TEM) as described (Hitachi, Ltd., Tokyo, Japan). The distribution of exosome particle size was measured by Flow NanoAnalyzer system (Xiamen, China) following the manufacturer's protocols, a kind of high sensitivity flow cytometry for nanoparticle analysis.

### Western blot

Exosomes were lysed in RIPA buffer and concentrations of protein were detected using BCA protein assay kit (Tiangen) according to the manufacturer's protocol. Protein samples loading into 10% polyacrylamide gels were separated by electrophoresis and then transferred to polyvinylidene difluoride (PVDF) membranes. After blocking with 5% skim milk, membranes were incubated overnight at 4 °C with the following specific primary antibodies: anti-CD63 (Abcam, ab193349, 1/2000), anti-TSG101 (ABclonal, A1692, 1/1000), anti-β-Tubulin (CST, 2128S, 1/2000). Among them, CD63 and Tumor susceptibility gene 101 protein (TSG101) are membranous and intraluminal markers of exosomes respectively, and β-Tubulin is a negative marker of exosomes. Afterwards, membranes were incubated with horseradish peroxidase (HRP)-conjugated secondary antibodies for 2 h at room temperature. Three washes (10 min/each) were done before chemiluminescent detection by Tanon luminescent imaging system (Shanghai, China).

### RNA extraction

TRIzol LS reagent (Life Technologies) was used to extracted total exosomal RNA following the manufacturer's protocols. Briefly, 750 μL TRIzol LS reagent added into 250 μL exosome particles suspension and incubated for 15 min prior to chloroform extraction. RNA degree glycogen (Life Technologies) was added as a carrier. After being washed by 75% alcohol two times, RNA pellets were re-suspended in 10 μL of RNase-free water and then microRNA Qubit kit and Qubit 3.0 (Thermo Fisher Scientific) were used to detect the concentration of exosomal small RNA per the manufacturer's protocols. The average yield of exosomal RNAs is approximately 40 ng/sample.

For RNA extraction from tissue, 0.1 g tissue sample was ground in liquid nitrogen and 1 ml TRIzol reagent (Life Technologies) was added into grinded tissue sample. Afterwards, sample was pipetted to a new tube and vibrated for 10 min. The following steps were complied with the manuscript protocols. Nanodrop 2000 (Thermo Fisher Scientific) was used to evaluate the quality and quantity of RNAs.

### Small RNA sequencing and reads mapping

The exosomal small RNA sequencing was performed by GUANGZHOU RIBOBIO CO., LTD (Guangzhou, China). The amount of 20 ng RNA per sample was used for small RNA library preparation in the sequencing. Sequencing libraries were generated using NEBNext^®^ Multiplex Small RNA Library Prep Set for Illumina^®^ (Illumina, San Diego, CA) following manufacturer's recommendations and index codes were added to attribute sequences to each sample. After being amplified, DNA fragments corresponding to 140-160 bp (the length of small non-coding RNA plus the 3' and 5' adaptors) were recovered in 8 μL of DNase- and RNase-free water. Library quality was assessed on the Agilent Bioanalyzer 2100 system using DNA High Sensitivity Chips. Libraries were sequenced using Illumina HiSeq 2500 System and 50 bp single-end reads were generated. No less than 10 million clean small RNA reads were obtained per samples. Obtained sequences were aligned to human mature miRNAs downloaded from miRBase (Release 21). The miRNA profiling was normalized using reads per million (RPM) of mapped miRNA sequences. RPM = (number of reads mapping to miRNA /total number of reads mapping to miRNA) ×1,000,000.

### MiRNA quantification

Reverse transcript of exosomal miRNA was performed using miScript II RT kit (Qiagen). Quantity PCR of miRNA was performed using miScript SYBR Green PCR Kit (Qiagen) with the provided universal reverse primers and the miRNAs specific primer. Real-time qPCR was performed on a Bio-rad CFX96 real time PCR system. For candidate miRNAs testing, 10 ng of exosomal total RNA was diluted to 0.5 ng/μL with nuclease free water after reverse transcription to cDNA. Then, 1 μL cDNA product was used for 10 μL qPCR reaction containing 5 μL Qiagen SYBR green Master Mix, 2 μL nuclease free water, 1 μL universal downstream primer and 1 μL miRNA specific upstream primer. The comparative cycle threshold (Ct) was used to evaluate the relative detection level of each miRNA in the sample. For internal reference determination, 10 ng exosomal small RNAs measured by microRNA Qubit assay were used for cDNA synthesis. To avoid heterogeneous residues especially alcohol from miRNA purification affecting reverse transcription efficiency and Ct value of the following qPCR, *C. elegans* miR-39-3p (cel-miR-39-3p, 1 × 10^7^ copies), a spike-in control, was added into samples to calibrate the following RT-qPCR steps. The calibrated method: Ct (calibrated) = Ct (miRNA) - Ct (miR-39) + Ct (average of miR-39). The calibrated Ct values of candidate references were analyzed with a web-based tool RefFinder to identify the most stable RNA as internal reference for exosomal miRNA quantitation 24*.*

Sequences of miRNAs specific primers:

hsa-miR-342-5p: ACACTCCAGCTGGGAGGGGTGCTATCTG

hsa-miR-574-5p: ACACTCCAGCTGGGTGAGTGTGTGTGTGTGAG

hsa-miR-222-3p: ACACTCCAGCTGGGAGCTACATCTGGCTAC

hsa-miR-425-5p: ACACTCCAGCTGGGAATGACACGATCACTC

hsa-miR-30c-5p: ACACTCCAGCTGGGTGTAAACATCCTACACTCTC

hsa-miR-16-5p: ACACTCCAGCTGGGTAGCAGCACGTAAATA

hsa-let-7a-5p: ACACTCCAGCTGGGTGAGGTAGTAGGTTGTATAG

hsa-miR-363-3p: ACACTCCAGCTGGGAATTGCACGGTATCC

hsa-miR-194-5p: ACACTCCAGCTGGGTGTAACAGCAACTCC

cel-miR-39-3p: ACACTCCAGCTGGGTCACCGGGTGTAAATCAGCTTG

U6 snRNA (RNU6): CTCGCTTCGGCAGCACA

### Statistical analysis

All statistical analyses were performed using GraphPad Prism 8 (GraphPad Software, La Jolla, CA, USA). For NGS data analysis, an unpaired two-sided *t*-test was applied with Benjamin-Hochberg multiple testing corrections to determine the differentially expressed miRNAs between LA-pre and HCs, and the paired two-sided Student's *t*-test was applied to analyze the differentially expressed miRNAs between LA-pre and LA-post. For RT-qPCR data, the paired two-sided Student's *t*-test was applied for analysis of LA-pre vs. LA-post, while the Mann-Whitney test was applied for LA-pre vs. HCs. The correlation between miRNA expression and gender or age of patients was analyzed by Pearson correlation. Receiver operating characteristic (ROC) curve was applied to assess the diagnostic power of candidate miRNAs. P values less than 0.05 were considered as statistically significant.

### Data Availability

The raw sequence data have been deposited in the Genome Sequence Archive in BIG Data Center, Beijing Institute of Genomics (BIG), Chinese Academy of Sciences, under accession number CRA001351 that is publicly accessible.

## Results

### Characterization of isolated exosomes and exosomal RNAs

**Figure 1:**
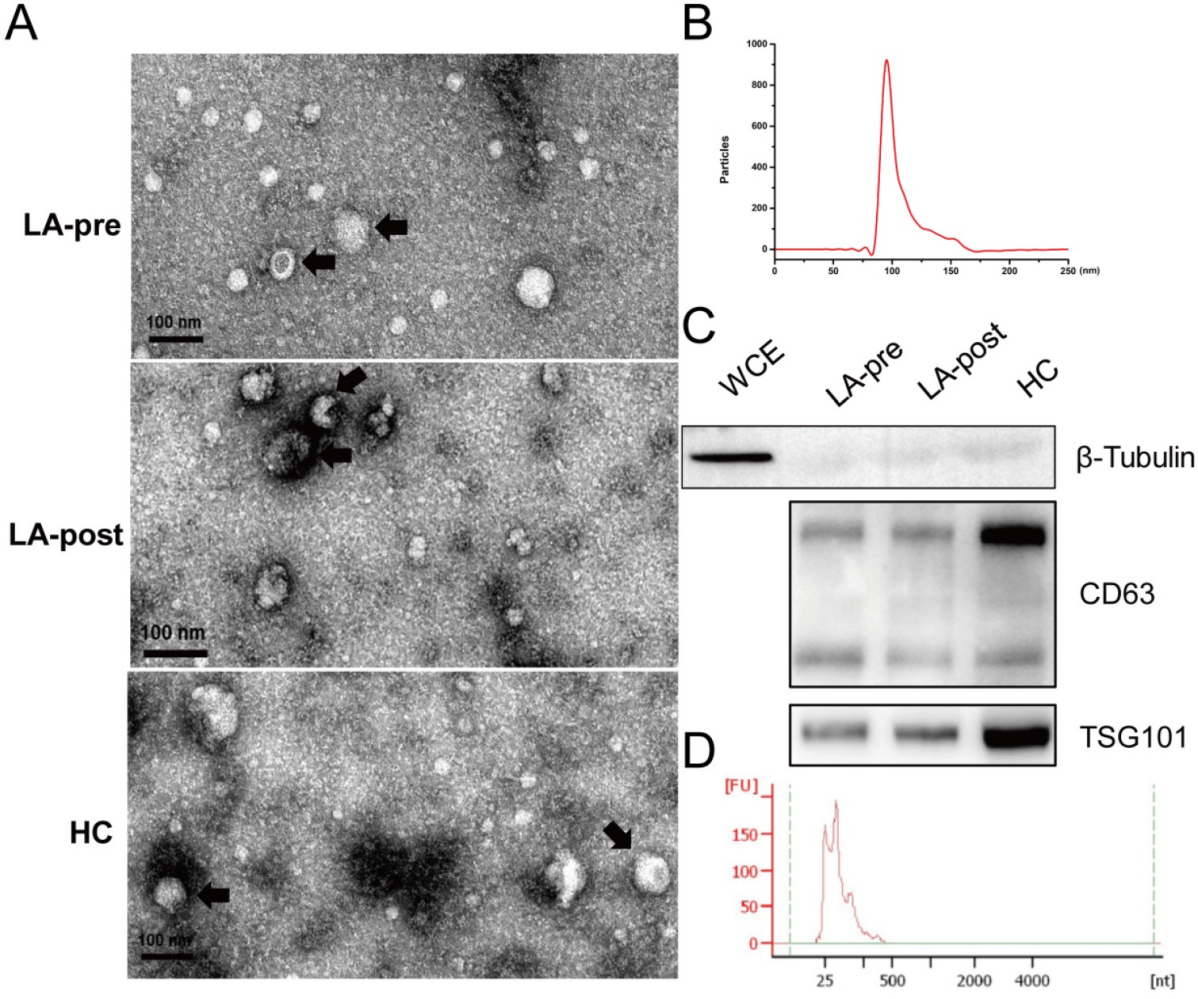
** Identification of circulating exosomes and exosomal RNAs.** (**A**) TEM observation of circulating exosomes from LA-pre, LA-post and HCs. Bars = 100 nm. (**B**) Particle size distribution of circulating exosomes calculated by Flow NanoAnalyzer software and the peak of the exosomal dimeter was 99.2 nm. (**C**) Western blot analysis of exosomal protein markers CD63 and TSG101 in circulating exosomes from LA-pre, LA-post and HCs. β-Tubulin is a negative marker of exosomes. Whole cell extracts (WCE) served as positive control of β-Tubulin. The main two bands of CD63 were detected. (**D**) Agilent Bioanalyzer 2100 analysis of the fragment lengths of exosomal total RNAs.

Exosome particles were isolated from plasma of peripheral blood by ultracentrifugation. TEM was used to identify the morphological features of exosome particles (Fig. 1A). Particle size of exosomes was analyzed by Flow NanoAnalyzer with the peak at 99.2 nm (Fig. 1B). Protein markers of exosomes (CD63 and TSG101) were tested by western blot (Fig. 1C). Then, exosomal total RNA was purified and the length of most exosomal RNA fragments was less than 200 nt (Fig. 1D), which agreed with previous reports 25. Concentrations of exosomal RNA couldn't be accurately measured by Nanodrop 2000 due to the abnormal 260/280 (< 1.70). Because we concerned small exosomal RNA fragments (miRNA), the microRNA Qubit kits and Qubit 3.0 were adopted to measure the concentration for the following RT-qPCR.

### Exosomal miRNAs profiles and data analysis

**Figure 2:**
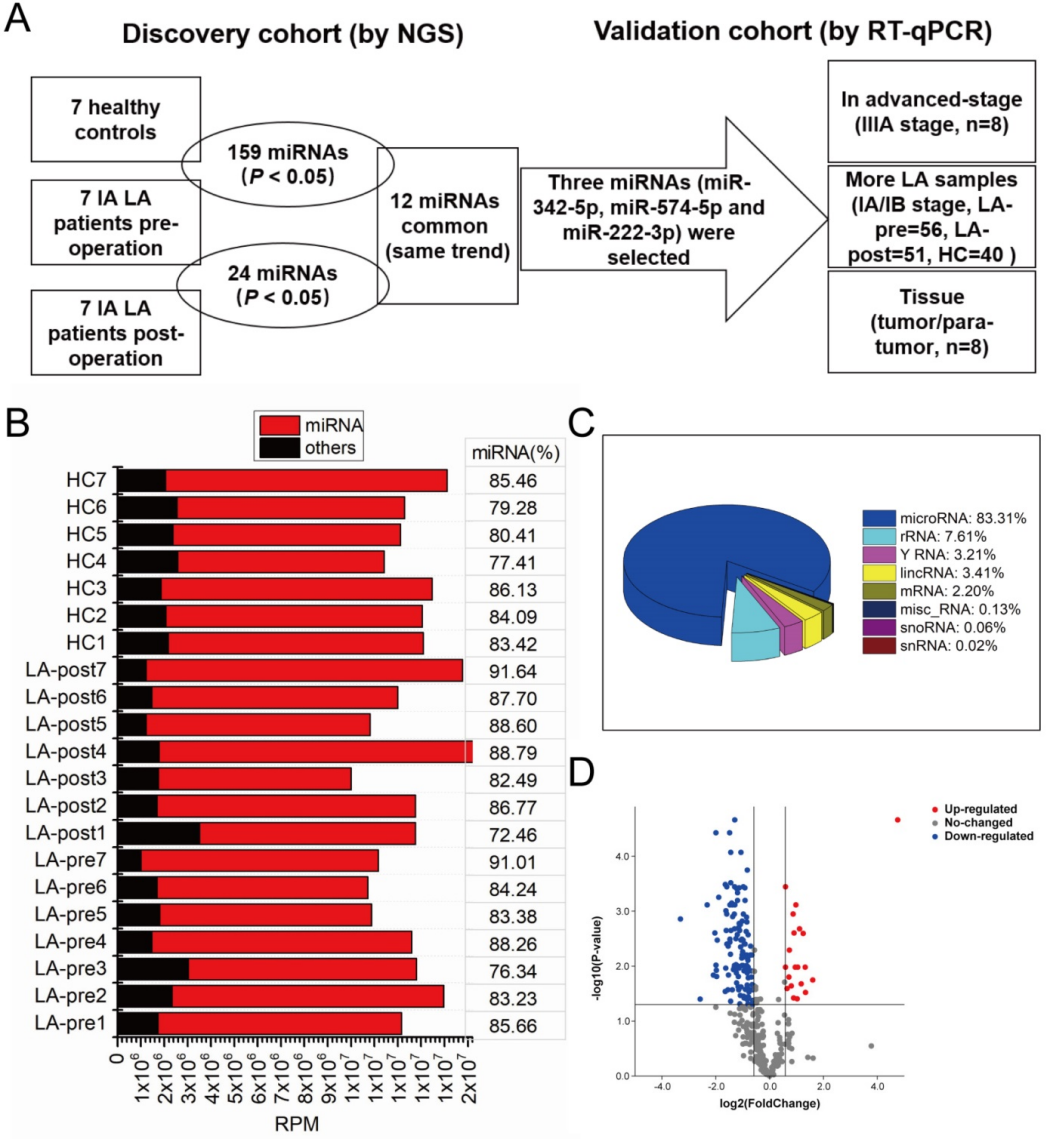
** Screening strategy and exosomal small RNA sequence.** (**A**) The flow chart of the screening circulating exosomal miRNAs for diagnosing early-stage LA. (**B**) Stack bar showing the number of miRNAs and other small RNA reads in circulating exosomes. MiRNA percentages in each sample were labeled. (**C**) Pie chart of the components of exosomal small RNA. (**D**) Volcano plot showing the differentially expressed exosomal miRNAs between LA-pre and HCs.

To discover diagnostic biomarkers of early-stage LA in circulating exosomal miRNAs (the screening strategy we used was shown in Fig. 2A), we performed small RNA sequencing of exosomal RNA samples derived from 7 LA patients (LA-pre and LA-post) and 7 HCs. Basic information of these samples was shown in Table 1. Greater than 10 million clean reads of each sample were obtained and the mapped miRNAs ranged between 72.46% and 91.64% (Fig. 2B), which agreed with previous reports 26. Based on the analysis of small RNA components, we also mapped small amount of rRNA, mRNA, sn/snoRNA, miscRNA and lincRNA along with major miRNA (Fig. 2C). RPM was used to quantify miRNA expression level. Compared with HCs, there were 159 miRNAs significantly dysregulated in LA-pre samples (adjusted P-value <0.05, Fig. 2D and Table S1). Compared with HCs, there were 24 miRNAs (10 down-regulated and 14 up-regulated) significantly dysregulated in LA-pre samples (P < 0.05, Table 2). We suggested that the 12 same trend miRNAs, which presented simultaneously in above two comparisons, could indirectly represent tumor-derived circulating exosomal miRNAs (Table 3).

### RNU6 was optimized internal reference in our study

RT-qPCR was applied to validate candidate miRNAs in extensive samples. Before performing RT-qPCR assay, selection of internal reference was inescapable due to the absent of standard internal reference in exosomal miRNA 27. We selected 3 miRNAs (miR-425-5p, miR-363-3p and miR-194-5p) stably expressed among different samples according to our sequencing data (Fig. S1A) then included 4 commonly used internal controls (RNU6, let-7a-5p, miR-16-5p and miR-30c-5p) to figure out the suitable internal reference RNA for our study. Six samples including two paired LA-pre/post and two HCs were used to test the internal controls by RT-qPCR. Cel-miR-39-3p was used as a spike-in control to calibrate the RT-qPCR efficiency (Fig. S1B). Based on a web-based tool RefFinder, RNU6 was the most stable one and the top-ranking reference RNA for this study (Fig. S1C).

### Candidate miRNAs validation by RT-qPCR

**Figure 3:**
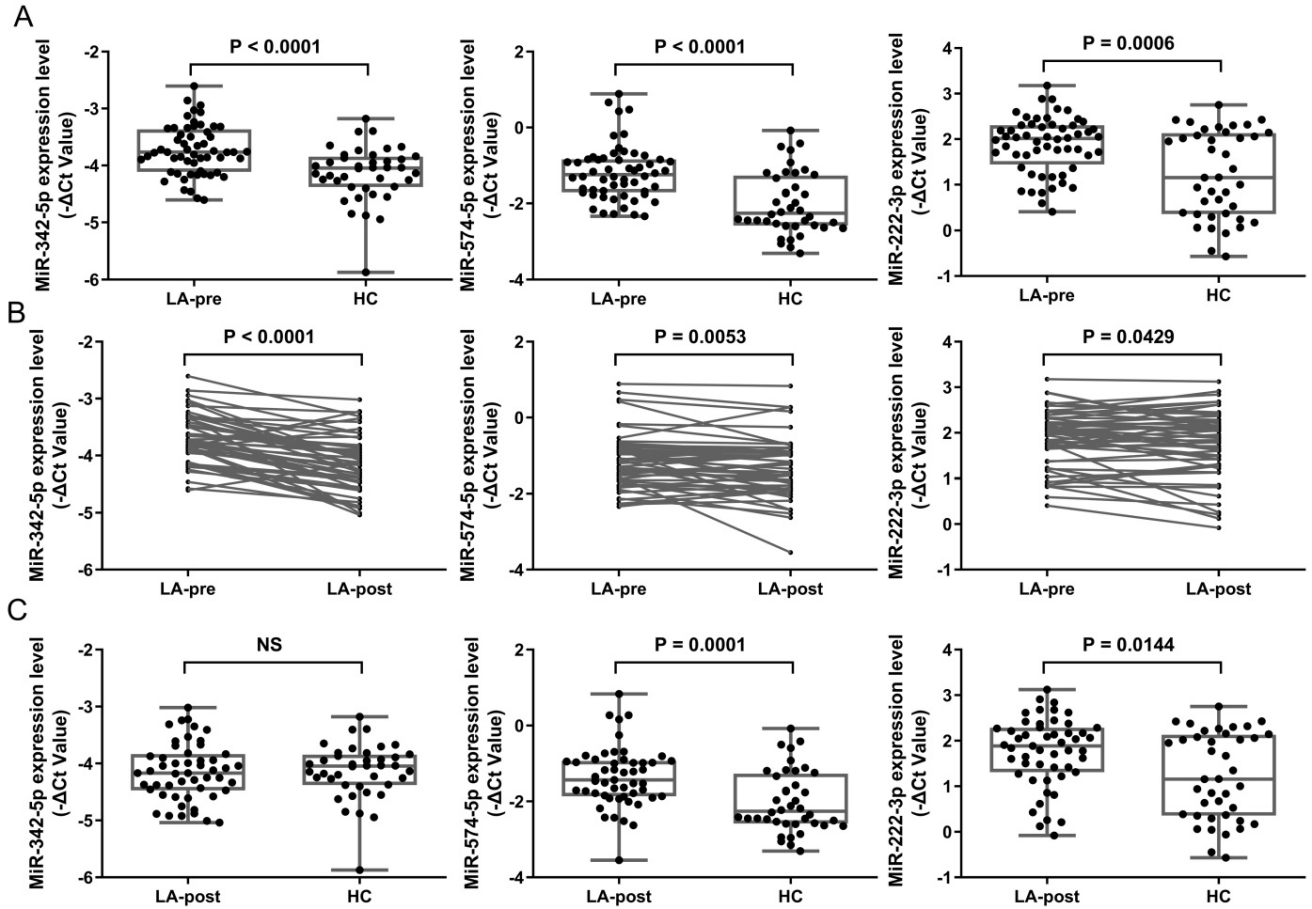
** RT-qPCR results of candidate miRNAs in early-stage LA patients.** (**A**) Scatter plots showing relative expression level of miR-342-5p, miR-574-5p and miR-222-3p in LA-pre (n = 56) and HCs (n = 40) samples. Statistical significance was analyzed by Mann-Whitney test. (**B**) Relative expression level of miR-342-5p, miR-574-5p and miR-222-3p in paired LA-pre and LA-post (n = 51). Statistical significance was analyzed by two-sided paired Student's *t*-test. (**C**) Scatter plots showing relative expression level of miR-342-5p, miR-574-5p and miR-222-3p in LA-post (n = 51) and HCs (n = 40) samples. Statistical significance was analyzed by Mann-Whitney test. Data were expressed as mean ± standard deviation (SD).

Considering the fold changes (FC) of miRNAs between LA-pre and LA-post, we chose the top 3 down-regulated miRNAs in LA-post group to do the following detection. We selected miR-342-5p, miR-574-5p and miR-222-3p from Table 3 as candidates to evaluate in ampliative validation cohort (56 LA patients in total, 51 paired LA-pre & -post and 5 LA-pre). Basic information of these samples was shown in Table 1. Exosomes were isolated from 4 ml plasma of each patient by ultracentrifugation. RNAs were extracted from exosome samples and diluted to 0.5 ng/μL for RT-qPCR. RNU6 was used as endogenous control. The average Ct values of each tested miRNA in validation samples was shown in Table S2. The RT-qPCR results showed that the expression levels of miR-342-5p (P < 0.0001), miR-574-5p (P < 0.0001) and miR-222-3p (P = 0.0006) were significantly higher in LA-pre (n = 56) than in HCs (n = 40) (Fig. 3A). For 51 paired LA-pre and LA-post samples analysis, miR-342-5p (P < 0.0001), miR-574-5p (P = 0.0053) and miR-222-3p (P = 0.0429) were significantly reduced after surgery (Fig. 3B). Furthermore, miR-342-5p showed no significant difference between LA-post and HCs (Fig. 3C), indicating that when tumor tissue was resected, circulating exosomal miRNA-342-5p returned to the level of HCs.

We did Pearson correlation analysis to detect the correlation of selected miRNAs with gender or age of patients, and found that the expression of miR-342-5p or miR-574-5p was not correlated with gender or age (data not shown).

We then detected these three miRNAs in advanced-stage (ⅢA) LA patients (n = 8) and found that only miR-342-5p was significantly decreased after tumor resection (Fig. S3A). Although miR-574-5p had the decreased trend, two-sided paired Student's *t*-test showed there was no significant with the P = 0.3082. (Fig. S3B). Regrettably, miR-222-3p seemed like a little increased after tumor resection (Fig. S3C).

### Evaluation of candidate miRNAs in tissue samples

**Figure 4:**
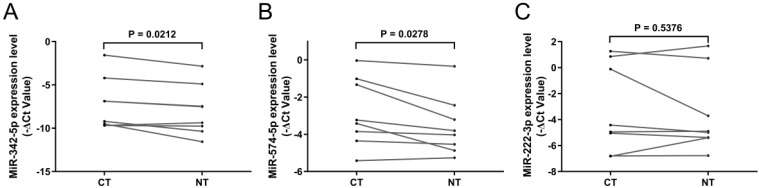
** RT-qPCR results of candidate miRNAs in tissue samples.** Relative expression level of (**A**) miR-342-5p, (**B**) miR-574-5p and (**C**) miR-222-3p in carcinoma tissue (CT) and adjacent non-cancerous tissue (NT, n = 8). Statistical significance was analyzed by two-sided paired Student's *t*-test.

To verify whether circulating exosomal miR-342-5p, miR-574-5p and miR-222-3p were mainly derived from carcinoma tissue, we analyzed their expressions in paired carcinoma tissue and adjacent non-cancerous tissue from 8 LA patients including 6 early-stages and 2 advanced-stages. It showed that the expressions of these miRNAs, except for miR-222-3p, were significantly upregulated in carcinoma tissue compared with adjacent non-cancerous tissue (Fig. 4A-C).

### Potential diagnostic value of candidate miRNAs

**Figure 5:**
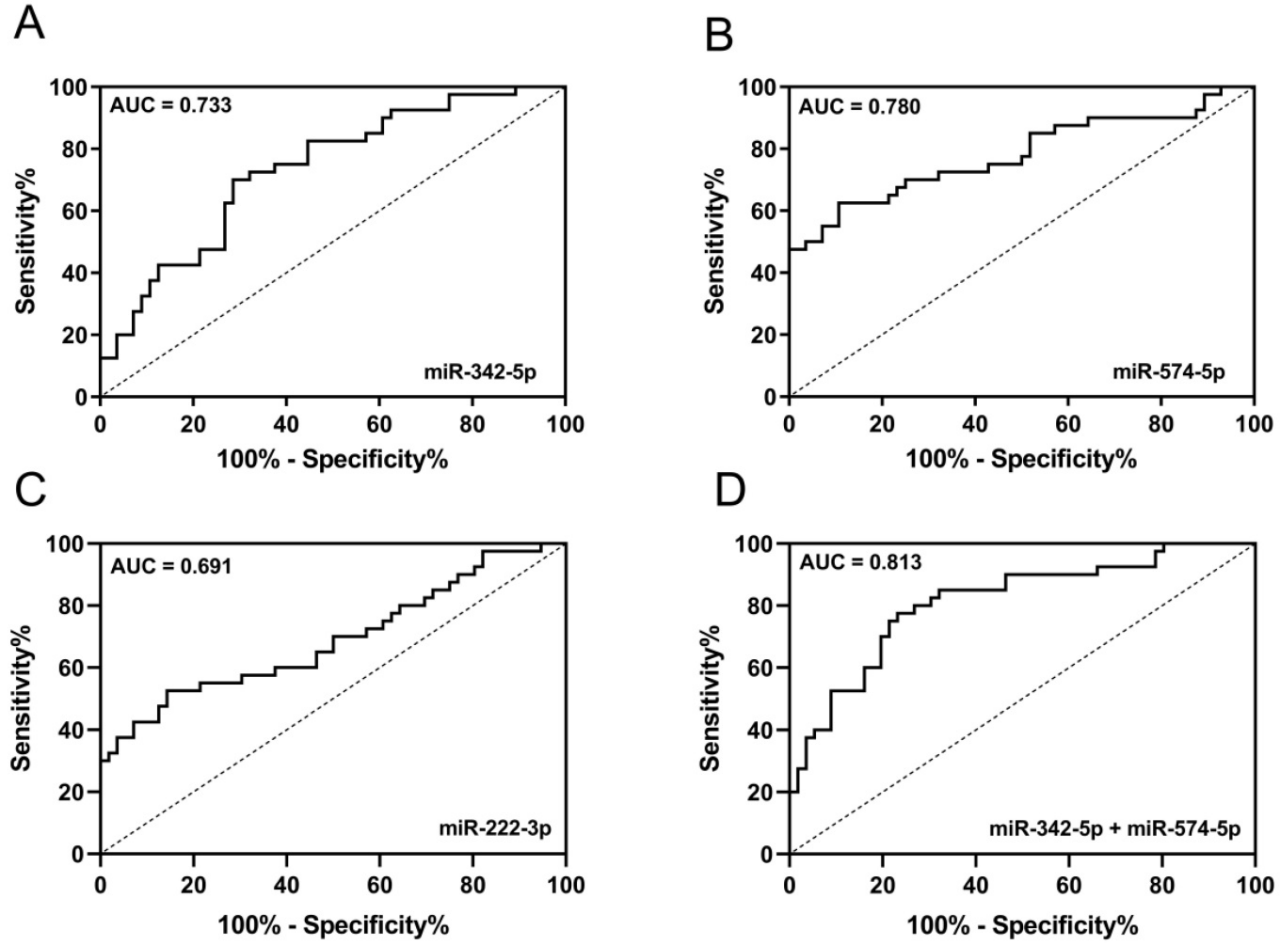
** The diagnostic potential of candidate miRNAs.** ROC curve analysis of (**A**) miR-342-5p, (**B**) miR-574-5p and (**C**) miR-222-3p to differentiate LA-pre patients (n = 56) and HCs (n = 40). (**D**) ROC curve analysis of combining miR-342-5p and miR-574-5p to differentiate LA-pre patients (n = 56) and HCs (n = 40).

ROC was used to evaluate the diagnostic performance of candidate miRNAs in discriminating early-stage LA patients from HCs. AUC of miR-342-5p, miR-574-5p and miR-222-3p were 0.733 (95% CI: 0.6323 to 0.8329), 0.780 (95% CI: 0.6792 to 0.8807), 0.691 (95% CI: 0.5795 to 0.8027) respectively (Fig. 5A-C). Since miR-342-5p and miR-574-5p were both increased in circulating exosomes and carcinoma tissue of LA patients, we tended to believe these two miRNAs were diagnostic biomarkers of early-stage LA. Then, we combined miR-342-5p and miR-574-5p to generate ROC curve and obtained with the AUC of 0.813 (95% CI: 0.7249 to 0.9009) with sensitivity and specificity of 80.0% and 73.2% respectively (Fig. 5D). In summary, the newly identified circulating exosomal miR-342-5p and miR-574-5p can distinguish early-stage LA patients from HCs with high sensitivity and specificity.

### Pathway analysis of miR-342-5p and miR-574-5p target genes

TargetScan Human version 7.0 was employed to predict the target genes of miR-342-5p and miR-574-5p. Because the predicted target genes with the lowest context score indicate the most confidence level, we filtered them with a context score threshold of < 0. Then the filtered target genes were subjected to Kyoto Encyclopedia of Genes and Genomes (KEGG) pathway analysis. After discarding the pathway with P ≥ 0.05, we obtained 57 pathways from miR-342-5p target genes and 28 pathways from miR-574-5p target genes respectively, which included several cancer-associated pathways such as cAMP signaling pathway, Pathways in cancer, Calcium signaling pathway and Non-small cell lung cancer and so on (Fig. S4).

## Discussion

Exosomes derived from tumor cells contain tumor specific medium that play diverse roles in tumor progression. More circulating exosomes were detected in tumor burden individuals than healthy individuals, suggesting that tumor cells secreted more exosomes 28. Circulating exosomal miRNAs have been studied in various cancers as diagnostic and prognostic biomarkers. Most studies only focused on the differentially expressed circulating exosomal miRNAs between individuals with tumor burden and without tumor burden. In our study, we analyzed circulating exosomal miRNAs not only between LA-pre and HCs, but also between LA-pre and the paired LA-post. Comparing LA-pre and paired LA-post samples could screen the tumor-specific exosomal miRNA, especially the down-regulated miRNAs after surgery 23. Theoretically, when tumor tissue is resected, tumor specific exosomes will gradually disappear from circulating exosomes. Our LA-post samples were collected in the day 3 after surgery. Therefore, tumor specific exosomes in blood could be adequately metabolized during the 3 days. However, the trauma of surgery may lead to some additional interference, especially from immune system. To target tumor-derived circulating exosomal miRNAs, we did not only compare the exosomal miRNA profiles of cancer patients to healthy controls, but also compare the ones before and after surgery. Therefore, we believe the candidate biomarkers were more likely derived from tumor because they were not only abnormally expressed in patients, but also sensitive to the presence or not of tumor in patients. When the tumor was removed, the amount of miRNA was lessened. Considering the fold change, we selected the top 3 upregulated miRNAs (miR-342-5p, miR-574-5p and miR-222-3p) in LA-pre compared to LA-post and HCs groups to validate in an enlarged cohort. According to the following RT-qPCR, circulating exosomal miR-342-5p and miR-574-5p were significantly elevated in LA patients and depressed after tumor resection. In addition, the elevated expression of these two miRNAs in carcinoma tissue was demonstrated in our study, further indicated that circulating exosomal miR-342-5p and miR-574-5p were mainly derived from carcinoma tissue.

Here, we reported that circulating exosomal miR-342-5p and miR-574-5p were promising diagnostic biomarkers of early-stage LA patients. Although there was no report about exosomal miR-342-5p in lung cancer, the reduced expression of exosomal miR-342-5p/3p in plasma has been demonstrated as a biomarker for Alzheimer disease 29. Moreover, circulating miR-342-5p has been found as a biomarker of several diseases like pertussis and atrial fibrillation 30, 31. When comparing the miRNA expression of lung cancer patients according to their ages, miR-342-5p was more highly expressed in younger patients (≤50 years) than it was in the elderly (>50 years) 32. Based on our small RNA sequencing data and RT-qPCR results, miR-574-5p did not recover to the level of HCs after tumor resection. We guessed the reason is that other cells, especial immune and inflammatory cells, also secrete exosomal miR-574-5p. However, miR-574-5p was significantly depressed both in LA-post and HCs when compared with LA-pre. Moreover, miR-574-5p from other source has been reported as a biomarker for lung cancer in several studies. The elevated serum miR-574-5p has been reported as a biomarker for early-stage NSCLC, which is corresponding with our results 33. Another report demonstrated that miR-574-5p were higher in lung cancer tissue compared with adjacent tissue of tumor 34. In the study of small cell lung cancer, miR-574-5p was higher expressed in tumor samples with chemo-resistance 35. Comparing advanced NSCLC patients with metastasis and non-metastasis, miR-574-5p was overexpressed in both serum and tumor tissue 36. For the mechanism studies, miR-574-5p was reported to facilitate tumor invasion and metastasis by targeting protein tyrosine phosphatase receptor type U (PTPRU), and promoted proliferation by influencing TLR9 signaling 34, 37.

Although the diagnostic technology has been in the rapid development, it is still quite urgent to improve the diagnostic accuracy at the early-stages of LA. The traditional clinical diagnostic technologies, such as X-ray and CT, showed an unsatisfactory outcome. About the circulating protein biomarkers used in clinical practice, they also lacked sufficient sensitivity and specificity. According to our patients' information, the CEA positive ratio was 8.9% and the CYFRA21-1 positive ratio was 12.0% (data not shown). Exosomes have been demonstrated as a new sample source of liquid biopsy in last decade. Plasma is a kind of conventional sample in clinical diagnosis, which means exosomes circulating in plasma are easy to obtain. Moreover, plasma has more abundant exosomes than other body fluids. Circulating exosomal miRNAs as the diagnostic markers have a broad prospect. The future study should focus on improving the accuracy of exosomal miRNA detection method.

In conclusion, circulating exosomal miR-342-5p and miR-574-5p can distinguish early-stage LA patients from HCs with high sensitivity and specificity that have potential to serve as promising diagnostic biomarkers for early-stage (ⅠA/ⅠB) LA patients. For clinical diagnostic application, further validation in external samples is needed.

## Supplementary Material

Supplementary figures and tables.

## Figures and Tables

**Table 1:** Clinical characteristics of patients and healthy controls

Variables	Circulating exosome sample						Tissue sample
	Discovery cohort (NGS)		Validation cohort (RT-qPCR)			Advanced-stage	
	LA-pre & -post	HCs	LA-pre	LA-post	HCs	LA-pre & -post	
Total number	7	7	56	51	40	8	8
Gender (Male/ Female)	4/3	4/3	20/36	18/33	12/28	4/4	3/5
Age (year, mean ± SD)	55.1±7.3	56±1.5	57.4±9.4	57.6±9.0	59.7±8.0	59.3±10.3	65.9±7.0
Clinical stage							
IA/IB	7	7	56	51	—	—	6
IIIA	—	—	—	—	—	8	2

**Table 2:** Exosomal miRNAs with significant variation between LA-pre and LA-post

Down-regulated miRNAs after tumor resection (P < 0.05)	Up-regulated miRNAs after tumor resection (P < 0.05)
hsa-miR-628-5p	hsa-miR-361-5p
hsa-miR-574-5p	hsa-miR-548o-3p
hsa-miR-2110	hsa-miR-107
hsa-miR-340-3p	hsa-miR-223-3p
hsa-miR-3158-3p	hsa-miR-338-5p
hsa-miR-342-5p	hsa-miR-132-3p
hsa-miR-222-3p	hsa-miR-182-5p
hsa-miR-3187-3p	hsa-miR-582-3p
hsa-miR-320c	hsa-miR-202-5p
hsa-miR-4489	hsa-miR-450b-5p
	hsa-miR-26b-3p
	hsa-miR-23a-3p
	hsa-miR-143-3p
	hsa-miR-556-5p

**Table 3:** Exosomal miRNAs with same trend and significant expression difference in LA-pre vs. LA-post and LA-pre vs. HCs

miRNA ID	Average of RPM			LA-pre vs. -post		LA-pre vs. HCs	
	LA-pre	LA-post	HCs	FC	P-value	FC	P-value
hsa-miR-342-5p	56.43	37.33	29.16	1.5115	0.0306	1.9353	0.0041
hsa-miR-574-5p	65.81	51.58	2.43	1.2761	0.0114	27.0889	0.0006
hsa-miR-222-3p	415.76	348.22	237.91	1.194	0.0397	1.7475	0.0111
hsa-miR-340-3p	38.13	32.47	16.06	1.1745	0.0229	2.3743	0.0006
hsa-miR-3158-3p	318.56	297.27	161.79	1.0717	0.0249	1.969	0.0006
hsa-miR-361-5p	12.08	13.68	27.61	0.8831	0.0014	0.4376	0.0006
hsa-miR-26b-3p	3.85	4.53	6.93	0.8503	0.0362	0.5554	0.0023
hsa-miR-450b-5p	4.15	4.99	12.84	0.8311	0.0354	0.3233	0.0006
hsa-miR-107	5.18	6.49	25.84	0.7976	0.0111	0.2004	0.0006
hsa-miR-132-3p	5.75	7.23	8.91	0.7953	0.0178	0.6452	0.0175
hsa-miR-548o-3p	8.13	10.71	11.58	0.7588	0.0071	0.7021	0.0262
hsa-miR-23a-3p	72.4	100.03	202.66	0.7238	0.039	0.3573	0.0006

## Abbreviations

AUC: area under the curve
CEA: carcinoembryonic antigen
CT: computerized tomography
EVs: extracellular vesicles
HCs: heathy controls
LA: lung adenocarcinoma
LA-post: lung adenocarcinoma of post-operation
LA-pre: lung adenocarcinoma of pre-operation
LSCC: lung squamous cell carcinoma
NGS: next generation sequence
NSCLC: non-small cell lung cancer
RPM: reads per million
ROC: receiver operating characteristic
RT-qPCR: reverse transcription-quantitative PCR
TEM: transmission electron microscopy
