# Supplementary material for: Tumor-derived circulating exosomal miR-342-5p and miR-574-5p as promising diagnostic biomarkers for early-stage Lung Adenocarcinoma
Source: Int J Med Sci. 2020 Jun 6;17(10):1428–38. doi: 10.7150/ijms.43500 (PMC7330662; doi:10.7150/ijms.43500)
Supplement: Supplementary file 1 — Supplementary figures and tables. [file ijmsv17p1428s1.pdf]

Tumor-derived Circulating Exosomal miR-342-5p And miR-574-5p As Novel Promising Diagnostic Biomarkers For Early-stage Lung Adenocarcinoma

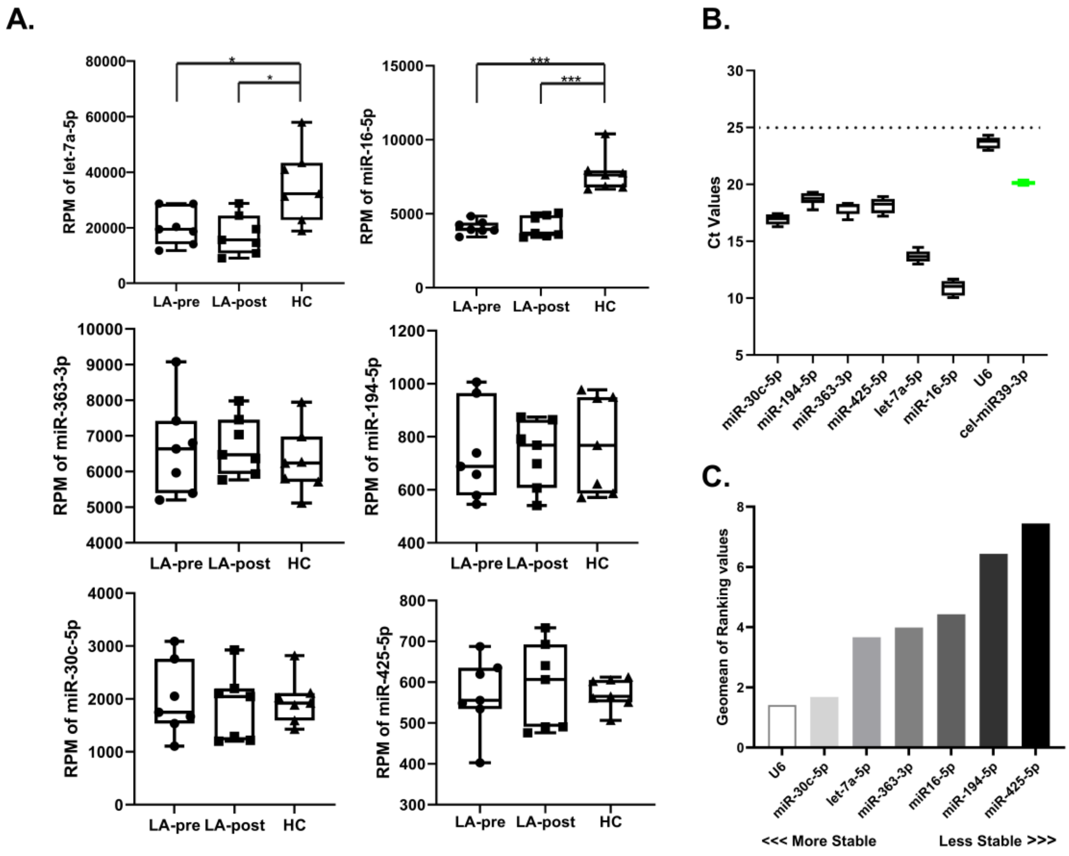

Figure S1. Selection of stable internal reference. (**A**) The expression level of selected six candidate reference miRNAs in sequencing data (*P < 0.05, ***P < 0.001). (**B**) Ct values of tested candidate internal references. (**C**) Histogram showing stability of candidate internal references analyzed by RefFinder. The less the geomean of ranking value is, the more stable the internal reference is.

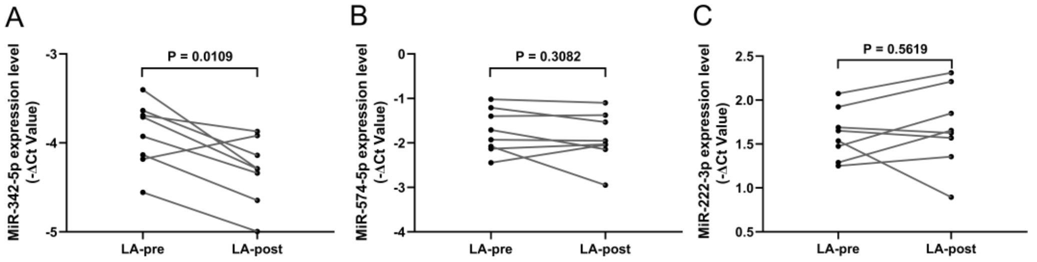

Figure S2. RT-qPCR results of candidate miRNAs in advanced-stage LA patients. Relative expression level of (**A**) miR-342-5p, (**B**) miR-574-5p and (**C**) miR-222-3p in paired LA-pre and LA-post (IIIA stage, n = 8). Statistical significance was analyzed by two-sided paired Student's *t*-test.

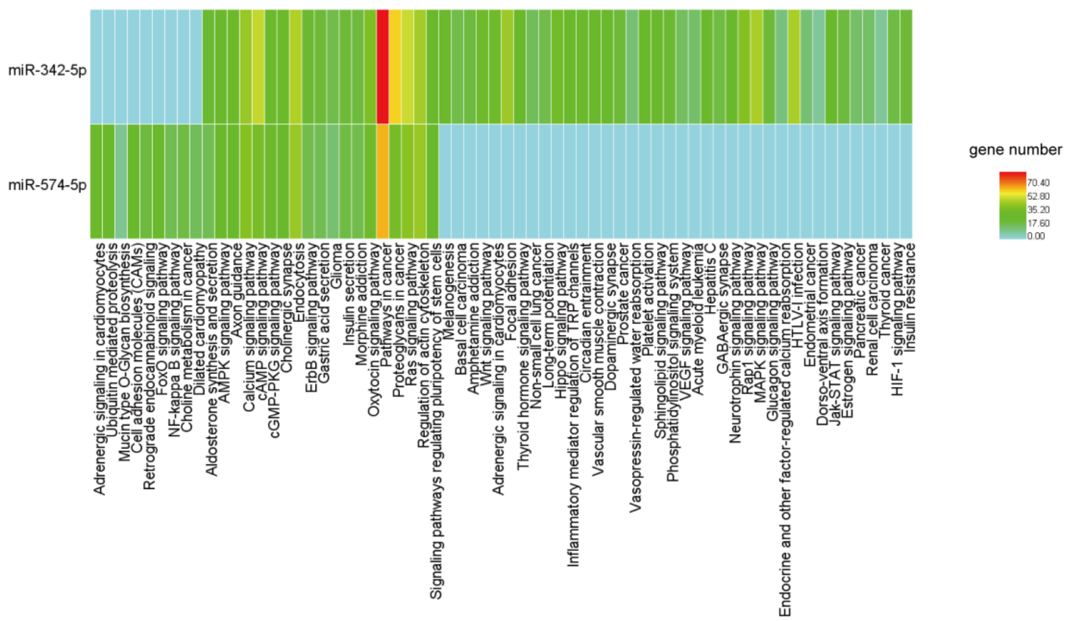

Figure S3. Heat map showing associated KEGG pathways of miR-342-5p and miR-574-5p predicted target genes.

Table S1. Differentially expressed circulating exosomal miRNAs in LA-pre and HC

miRNA	Average RPM of LA-pre	Average RPM of HC	FC	Adjusted P-value
hsa-miR-144-5p	1249	3065	0.4076	<0.0001
hsa-miR-574-5p	65.81	2.43	27.0889	<0.0001
hsa-miR-103a-3p	2300	9163	0.2511	<0.0001
hsa-miR-23a-3p	72.4	202.7	0.3573	<0.0001
hsa-miR-27b-3p	239.4	500.5	0.4783	<0.0001
hsa-miR-3912	2.909	7.915	0.3676	<0.0001
hsa-miR-548k	6.987	12.41	0.5630	0.0002
hsa-miR-221-3p	94.43	256.5	0.3681	0.0003
hsa-miR-185-5p	3144	9832	0.3198	0.0003
hsa-miR-3140-3p	1.085	2.134	0.5083	0.0004
hsa-miR-548av-5p	3.554	8.737	0.4068	0.0004
hsa-miR-451a	582925	386853	1.5068	0.0004
hsa-miR-374b-3p	0.9535	2.872	0.3320	0.0004
hsa-miR-17-5p	803.3	1762	0.4559	0.0004
hsa-miR-16-5p	4082	7711	0.5294	0.0004
hsa-miR-500a-3p	27.82	65.06	0.4276	0.0004
hsa-miR-484	323.9	739.6	0.4380	0.0005
hsa-miR-190a	5.522	20.46	0.2699	0.0006
hsa-let-7g-5p	30374	57165	0.5313	0.0006
hsa-miR-361-5p	12.08	27.61	0.4376	0.0006
hsa-miR-22-3p	1212	3217	0.3767	0.0007
hsa-miR-3158-3p	318.6	161.8	1.9690	0.0008
hsa-miR-548ay-5p	23.6	57.79	0.4084	0.0008
hsa-miR-590-3p	7.487	20.79	0.3601	0.0008
hsa-miR-107	5.178	25.84	0.2004	0.0008
hsa-miR-93-5p	1111	2901	0.3831	0.0008
hsa-miR-502-3p	38.1	117.3	0.3248	0.001
hsa-miR-651	13.49	40.7	0.3314	0.0011
hsa-miR-1180	115	62.77	1.8324	0.0011
hsa-miR-598	12.61	24.78	0.5090	0.0011
hsa-miR-21-5p	4028	9986	0.4033	0.0011
hsa-miR-15a-5p	43.05	111.4	0.3863	0.0013
hsa-miR-150-5p	168.4	304.7	0.5527	0.0013
hsa-miR-1	1.069	10.62	0.1006	0.0014
hsa-miR-181a-5p	422.7	857	0.4932	0.0015
hsa-miR-146b-5p	406.4	729.6	0.5570	0.0016
hsa-miR-190b	2.52	5.516	0.4568	0.0017
hsa-miR-130b-3p	6.463	13.77	0.4694	0.0017
hsa-miR-378g	1.89	4.011	0.4711	0.0017

hsa-miR-548at-5p	0.6231	1.381	0.4511	0.002
hsa-miR-148a-3p	13847	6404	2.1622	0.0021
hsa-miR-339-3p	1.878	4.626	0.4061	0.0021
hsa-miR-4521	1.852	5.017	0.3691	0.0022
hsa-miR-199b-3p	37.41	114.3	0.3272	0.0022
hsa-miR-4306	416.1	898.9	0.4629	0.0022
hsa-miR-148b-3p	966.8	1765	0.5479	0.0023
hsa-miR-30e-5p	766.9	3138	0.2444	0.0025
hsa-miR-659-5p	7.024	3.745	1.8757	0.0025
hsa-miR-340-3p	38.13	16.06	2.3743	0.0025
hsa-miR-221-5p	6.485	14.91	0.4350	0.0027
hsa-miR-421	24.76	43.07	0.5750	0.0027
hsa-miR-20a-5p	1235	2942	0.4198	0.0032
hsa-miR-152	23.08	47.05	0.4904	0.0032
hsa-miR-199b-5p	6.704	18.64	0.3597	0.0032
hsa-miR-185-3p	51.02	104.9	0.4866	0.0033
hsa-miR-20b-5p	275.5	545.2	0.5054	0.0034
hsa-miR-126-5p	100.3	385.9	0.2598	0.0034
hsa-miR-342-3p	15.39	32.09	0.4795	0.0034
hsa-miR-374a-3p	34.29	95.27	0.3599	0.0037
hsa-miR-106b-5p	272.1	818.4	0.3325	0.004
hsa-miR-378a-3p	380.6	715.6	0.5318	0.004
hsa-let-7f-5p	31855	61073	0.5216	0.004
hsa-miR-576-3p	12.1	35.17	0.3442	0.0043
hsa-miR-98-5p	504.5	822.3	0.6135	0.0043
hsa-miR-106a-5p	82.76	174.5	0.4742	0.0045
hsa-miR-362-5p	9.323	13.76	0.6776	0.0051
hsa-miR-330-5p	10.98	6.599	1.6643	0.0051
hsa-miR-374a-5p	362.3	724.6	0.5000	0.0055
hsa-miR-660-5p	65.09	178.3	0.3650	0.0061
hsa-miR-26b-3p	3.851	6.934	0.5554	0.0063
hsa-miR-652-3p	448.6	707.2	0.6343	0.0063
hsa-miR-532-5p	748.5	1230	0.6085	0.0063
hsa-miR-186-5p	1402	2844	0.4928	0.0063
hsa-miR-454-3p	151.7	274	0.5535	0.0063
hsa-miR-200c-3p	7.307	12.72	0.5744	0.0068
hsa-miR-548e	5.097	10.18	0.5009	0.0068
hsa-miR-93-3p	18.12	34.45	0.5260	0.0068
hsa-miR-29b-2-5p	1.906	3.901	0.4886	0.0071
hsa-miR-324-3p	38.48	67.24	0.5722	0.0078
hsa-let-7d-5p	2482	5901	0.4205	0.0094
hsa-miR-340-5p	81.04	322.4	0.2513	0.0096
hsa-miR-210	28.87	58.83	0.4908	0.0097

hsa-miR-101-3p	5838	14426	0.4047	0.0097
hsa-miR-146a-5p	306	707.6	0.4325	0.0097
hsa-miR-26a-5p	12902	23324	0.5532	0.0097
hsa-miR-624-5p	5.307	10.52	0.5045	0.0097
hsa-miR-21-3p	3.81	9.409	0.4049	0.0101
hsa-miR-184	2.856	1.143	2.4996	0.0104
hsa-miR-16-2-3p	41.1	19.93	2.0626	0.0104
hsa-miR-151a-3p	3724	2472	1.5065	0.0104
hsa-miR-342-5p	56.43	29.16	1.9353	0.0104
hsa-miR-18a-5p	23.81	42.06	0.5662	0.0104
hsa-miR-28-3p	36.51	63.39	0.5760	0.0104
hsa-miR-19a-3p	59.26	147.3	0.4024	0.0104
hsa-miR-4446-3p	0.4439	1.055	0.4207	0.0107
hsa-miR-548ah-3p	1.012	2.227	0.4546	0.0109
hsa-miR-450b-5p	4.15	12.84	0.3233	0.0109
hsa-let-7c	75.43	141.9	0.5316	0.0113
hsa-miR-378f	1.126	2.861	0.3937	0.0115
hsa-miR-144-3p	1026	4040	0.2539	0.0119
hsa-miR-766-3p	1.879	3.224	0.5827	0.0122
hsa-miR-425-3p	6.894	13.34	0.5169	0.0122
hsa-miR-130a-3p	24.14	38.51	0.6268	0.0122
hsa-miR-939-5p	2.383	3.521	0.6769	0.0124
hsa-miR-29a-3p	34.09	80.27	0.4247	0.0133
hsa-miR-140-3p	1938	3451	0.5615	0.0133
hsa-miR-4286	2.383	6.872	0.3468	0.0145
hsa-miR-19b-3p	115.4	490.5	0.2352	0.0146
hsa-miR-142-5p	119.6	467.6	0.2557	0.0155
hsa-miR-18b-5p	0.483	1.08	0.4472	0.0155
hsa-miR-99a-5p	149.4	90.92	1.6438	0.0158
hsa-miR-664a-5p	2.099	3.346	0.6273	0.0158
hsa-miR-374b-5p	411.2	712.4	0.5772	0.0163
hsa-miR-3656	7.603	2.503	3.0374	0.0179
hsa-miR-581	5.833	3.962	1.4723	0.0195
hsa-miR-1273c	0.8208	1.921	0.4274	0.0202
hsa-miR-1306-3p	6.353	2.813	2.2582	0.021
hsa-miR-320b	107.1	167.7	0.6388	0.0217
hsa-miR-25-5p	7.745	13.8	0.5613	0.0221
hsa-miR-1976	8.176	12.36	0.6613	0.0227
hsa-miR-222-3p	415.8	237.9	1.7475	0.0229
hsa-let-7e-5p	8.268	18.58	0.4450	0.0237
hsa-miR-3200-5p	3.545	7.401	0.4791	0.0239
hsa-miR-155-5p	51.13	73.21	0.6984	0.0239
hsa-miR-1255b-5p	46.88	29.89	1.5684	0.0256

hsa-let-7i-3p	7.251	12.53	0.5787	0.0259
hsa-miR-106b-3p	237.9	340.8	0.6982	0.0265
hsa-miR-181c-5p	2.889	5.39	0.5360	0.0265
hsa-miR-3173-5p	0.9002	1.996	0.4509	0.027
hsa-miR-3176	1.037	3.032	0.3419	0.027
hsa-miR-224-5p	1.952	5.105	0.3823	0.027
hsa-miR-17-3p	7.03	10.92	0.6437	0.027
hsa-miR-301a-3p	0.7206	2.258	0.3191	0.0289
hsa-miR-532-3p	7.676	13.07	0.5872	0.0295
hsa-miR-5582-3p	2.165	0.8586	2.5216	0.0301
hsa-miR-335-5p	6.095	12.95	0.4708	0.0341
hsa-miR-450a-5p	12.77	24.99	0.5111	0.0352
hsa-miR-181d	1.88	3.236	0.5808	0.0368
hsa-miR-3200-3p	13.64	24.22	0.5629	0.0368
hsa-miR-1288	0.9254	0.4983	1.8571	0.038
hsa-miR-15b-5p	186.8	360.2	0.5185	0.038
hsa-miR-135a-5p	1.727	3.128	0.5521	0.0384
hsa-miR-501-3p	30.44	54.58	0.5577	0.0384
hsa-miR-29c-3p	16.1	30.14	0.5342	0.0384
hsa-miR-514a-3p	1.791	0.8799	2.0356	0.0394
hsa-miR-1307-5p	1.836	10.97	0.1673	0.0398
hsa-let-7i-5p	69571	87935	0.7912	0.0398
hsa-miR-30e-3p	552	739.4	0.7466	0.0405
hsa-miR-24-3p	521.3	413.6	1.2604	0.0405
hsa-miR-548o-3p	8.131	11.58	0.7021	0.0405
hsa-miR-345-5p	22.68	40.31	0.5626	0.0405
hsa-miR-5001-3p	1.642	2.311	0.7104	0.0414
hsa-miR-132-3p	5.75	8.913	0.6452	0.042
hsa-miR-874	0.7498	2.044	0.3668	0.0435
hsa-let-7a-3p	74.96	115.7	0.6477	0.0444
hsa-miR-454-5p	12.79	17.83	0.7170	0.0466
hsa-miR-941	79.2	170.6	0.4643	0.0487
hsa-miR-28-5p	11.33	17.65	0.6421	0.0488
hsa-let-7a-5p	20236	35337	0.5727	0.0489

Table S2. Average Ct values of candidate biomarkers detected in validation samples

miRNA	Mean±SD（Ct）		
	LA-pre(n=56)	LA-post(n=51)	HC（n=40）
hsa-miR-342-5p	25.96±0.90	26.66±0.97	26.64±0.98
hsa-miR-574-5p	23.41±0.89	23.87±0.92	24.47±0.87
hsa-miR-222-3p	20.35±1.16	20.76±1.36	21.24±0.99
RNU6	22.23±1.09	22.52±1.18	22.47±1.05
